# Melatonin Exerts Chondroprotective Effects Against Osteoarthritis by Promoting PI3K/AKT/FoxO3‐Mediated Mitophagy

**DOI:** 10.1002/kjm2.70131

**Published:** 2025-11-05

**Authors:** Chao Huang, Gang Zhang, Ying‐Kai Ma, Xin‐Nan Ma, Song‐Cen Lv

**Affiliations:** ^1^ Department of Orthopedics The Second Affiliated Hospital of Harbin Medical University Harbin China; ^2^ The Key Laboratory of Myocardial Ischemia, Harbin Medical University, Ministry of Education Harbin China; ^3^ Emergency Medicine Center (Emergency Surgery), affiliated Hospital of Guangdong Medical University Zhanjiang China

**Keywords:** chondrocytes, melatonin, mitophagy, osteoarthritis, phosphatidylinositol‐3 kinase/protein kinase B/forkhead box O3

## Abstract

Osteoarthritis (OA) is a prevalent degenerative joint disease. This study combines bioinformatics analysis with in vivo and in vitro experiments to elucidate the molecular mechanisms through which melatonin (MT) regulates mitophagy to alleviate OA. Rat and chondrocyte OA models were established via anterior cruciate ligament transection or interleukin (IL)‐1β induction, followed by treatment with MT, Cyclosporine A (a mitophagy inhibitor), and 740Y‐P (a phosphatidylinositol‐3 kinase [PI3K] activator). Pathological changes in cartilage, histological scores, and cell apoptosis were evaluated alongside chondrocyte viability, apoptosis, mitochondrial morphology, mitochondrial membrane potential, and mitophagy using H&E and Safranin O‐fast green staining, Osteoarthritis Research Society International scoring (OARSI), TUNEL staining, CCK‐8, flow cytometry, transmission electron microscopy, JC‐1 staining, and immunofluorescence. Levels of inflammatory factors and mitophagy‐related protein levels were determined by ELISA and western blot. Bioinformatics analysis was applied to investigate the regulatory mechanisms of MT on mitophagy in OA. In vivo, MT mitigated OA by enhancing mitophagy and reducing apoptosis of cartilage cells. In vitro, MT attenuated IL‐1β‐induced chondrocyte apoptosis through mitophagy activation, and this effect was partially reversed by mitophagy inhibition. Mechanistically, the PI3K/protein kinase B (AKT)/forkhead box O3 (FoxO3) axis appeared to play a central role. MT suppressed PI3K/AKT signaling, thereby upregulating FoxO3 expression and promoting mitophagy, ultimately reducing chondrocyte apoptosis. Collectively, these findings suggest that MT enhances mitophagy via inhibition of the PI3K/AKT pathway, and subsequent upregulation of FoxO3, leading to reduced apoptosis of cartilage cells and attenuation of OA progression in rats.

## 1 Introduction

Osteoarthritis (OA) is a degenerative disorder that affects the entire joint, primarily characterized by cartilage degradation resulting from abnormal mechanical stress on the joint or adjacent tissues [1]. It is the most prevalent joint disease and the leading cause of physical activity restriction in adults, with an estimated global prevalence of over 240 million individuals [2]. The pathogenesis of OA is multifactorial and includes reduced proliferation of chondrocytes and extracellular matrix (ECM) synthesis, along with increased chondrocyte apoptosis and ECM degradation [3]. Strategies that preserve cartilage integrity and prevent degeneration have demonstrated therapeutic benefit in OA [4]. However, current treatment options remain limited, as no pharmacologic agents have yet been approved as disease‐modifying therapies for OA [1]. This underscores the urgent need to elucidate the mechanism underlying OA progression and to identify effective therapeutic strategies.

Mitophagy, a selective form of autophagy, is the process by which autophagosomes specifically target and degrade damaged or depolarized mitochondria [5]. Dysregulated mitophagy plays a critical role in the pathogenesis of numerous degenerative diseases, including OA [6]. Enhancing mitophagy and restoring autophagic flux can attenuate oxidative stress‐related mitochondrial dysfunction and thereby limit chondrocyte apoptosis [7]. Increasing evidence suggests that mitophagy serves as a protective cellular mechanism in chondrocytes by removing dysfunctional mitochondria, reducing oxidative stress, inhibiting apoptosis, and ultimately slowing OA progression [8]. Nevertheless, the molecular mechanisms linking autophagy and OA remain incompletely understood.

Melatonin (N‐acetyl‐5‐methoxytryptamine, MT) is an indoleamine primarily secreted by the pineal gland, synthesized from tryptophan through a series of enzymatic reactions. MT possesses potent antioxidant and anti‐inflammatory properties [9]. Its receptors, MT receptors 1 (MT1) and 2 (MT2), are G protein‐coupled receptors located on both mitochondria and the cell membrane [10]. Notably, chondrocytes are capable of synthesizing small amounts of MT, and both endogenous and exogenous MT regulate cartilage growth and maturation via MT1 and MT2 signaling [11]. Recent studies have shown that MT attenuates inflammation, oxidative stress, and chondrocyte apoptosis, thereby preventing cartilage destruction and OA progression [12]. Moreover, MT has been demonstrated to prevent oxidative damage and preserve mitochondrial function across various cell types and in vivo models, establishing it as a crucial regulator of mitochondrial integrity and physiology [13]. Bioinformatics analysis from recent work suggests that the phosphatidylinositol‐3 kinase/protein kinase B/forkhead box O3 (PI3K/AKT/FoxO3) pathway may play an important role in MT‐mediated regulation of mitophagy. Additionally, MT suppresses the upregulation of mitochondrial NADPH oxidase 4, ameliorates mitochondrial dysfunction, and effectively delays ferroptosis, thereby alleviating OA [14]. However, limited studies have investigated whether MT mitigates OA through PI3K/AKT/FoxO3 pathway–dependent regulation of mitophagy. The present study aims to elucidate the effects and underlying molecular mechanisms of MT in OA, focusing on its regulation of PI3K/AKT/FoxO3 pathway‐mediated mitophagy. These findings may provide novel insights and a theoretical foundation for the development of effective OA therapies.

## 2 Materials and Methods

### 2.1 Ethics Statement

All animal experiments were reviewed and approved by the Medical Ethics Committee of The Second Affiliated Hospital of Harbin Medical University, approval number: YJSKY2022‐080. All procedures were performed in accordance with the approved protocol, with efforts made to minimize animal numbers and reduce suffering.

### 2.2 Animal Modeling and Grouping

Specific pathogen‐free grade male Sprague–Dawley rats (SYXK (Heilongjiang) 2023–002, 8 weeks old, weighing 200 ± 20 g, *n* = 72) were obtained from Harbin Zhongke Saines Biotechnology Co. LTD (Harbin, Heilongjiang, China). Animals were housed under standard conditions (12‐h light/dark cycle, temperature: 22°C–24°C, humidity: 60%) with free access to food and water. After a one‐week acclimatization period, osteoarthritis (OA) was induced in the right knee by anterior cruciate ligament transection (ACLT) (the ACLT group) [15]. Sham‐operated rats underwent incision of the joint capsule followed by immediate suturing without ligament transection. After surgery, all rats were allowed unrestricted cage activity.

Rats were randomly assigned to six groups (*n* = 12 per group): Sham group, OA group, MT group, MT + CsA group, MT + 740Y‐P group, and MT + Vehicle group.

Interventions: Beginning from Day 7 post‐surgery, rats received intra‐articular knee injections with 20 μL of MT (10 mg/mL, HY‐B0075, MCE, Shanghai, China) [16] and 2 μg/kg of a mitophagy inhibitor Cyclosporine A (CsA; HY‐B0579, MCE) [17], or 10 mg/kg of a PI3K activator 740Y‐P (HY‐P0175, MCE) [18], or an equal volume of Vehicle (10% dimethyl sulfoxide [DMSO], 40% polyethylene glycol 300, 5% Tween‐80, 45% saline). Rats in the Sham group received an injection of an equivalent volume of normal saline, twice a week (on the 1st and 4th days), for a total of 8 weeks. At the end of the 8‐week intervention, rats were anesthetized with 2% isoflurane and euthanized by cervical dislocation. Synovial fluid was collected from the knee joint, and the right knee was excised. Cartilage samples from six randomly selected rats per group were fixed in 4% paraformaldehyde (30525‐89‐4, Sigma‐Aldrich, St Louis, MO, USA). Cartilage from the remaining six rats per group was homogenized and preserved at −80°C for further analyses.

### 2.3 Safranin O‐Fast Green Staining and Histological Scoring

Cartilage samples were fixed in 4% paraformaldehyde for 7 days and subsequently decalcified in 20% ethylenediamine tetraacetic acid (pH 7.4). The decalcification solution was refreshed every 3 days until the tissues were softened. Samples were then dehydrated, embedded in paraffin, and sectioned at 5 μm thickness. For safranin O‐fast green staining, sections were dewaxed, rehydrated, and stained with fast green (G1053‐2, Servicebio, Wuhan, Hubei, China), then with safranine O (G1053‐1, Servicebio) for 5 min. Sections were rapidly dehydrated in anhydrous ethanol, cleared with xylene (5 min), and mounted with neutral gum. Images were acquired using an AH‐2 light microscope (Olympus, Tokyo, Japan). Histological grading was performed using the Pritzker's Osteoarthritis Research Society International (OARSI) scoring system [19].

### 2.4 Hematoxylin and Eosin (H&E) Staining

Paraffin‐embedded rat articular cartilage tissue wax blocks were sectioned at 5 μm thickness, dewaxed, and subjected to antigen retrieval. Sections were treated with 3% H_2_O_2_ (10011218, Sinopharm Chemical Reagent Co. Ltd., Shanghai, China) for 15 min to block endogenous peroxidase activity, followed by incubation with 1% bovine serum albumin (A602440‐0050, Sangon Biotech Co. Ltd., Shanghai, China) to reduce nonspecific antibody binding. Sections were then stained with H&E solution (H8070, Solarbio, Beijing, China). Histological changes were observed and imaged under a microscope (BX53, Olympus).

### 2.5 Terminal Deoxynucleotidyl Transferase‐Mediated dUTP Nick End‐Labeling (TUNEL) Staining

Apoptosis in articular cartilage tissue was assessed using a TUNEL cell apoptosis (red fluorescence) assay kit (C1089, Beyotime, Shanghai, China) that detects red fluorescence. The procedure was performed according to the manufacturer's instructions.

### 2.6 Enzyme‐Linked Immunosorbent Assay (ELISA)

Concentrations of interleukin (IL)‐6 (JL20268, Jonlnbio, Shanghai, China) and tumor necrosis factor‐α (TNF‐α) (JLW10484, Jonlnbio) in synovial fluid were measured using ELISA kits, following the manufacturers' protocols.

### 2.7 Cell Modeling and Grouping

Primary chondrocytes (PC‐078r, Saios Biological Technology Co. Ltd., Wuhan, Hubei, China) were cultured in Dulbecco's modified Eagle medium (Hyclone, Waltham, MA, USA) supplemented with 1% streptomycin, 1% penicillin (Hyclone), and 10% fetal bovine serum (Gibco, Thermo Scientific, Waltham, MA, USA). Cells were maintained at 37°C in a humidified incubator with 5% CO_2_. Upon reaching ~80% confluence, cells were passaged at a 1:3 ratio.

To ensure phenotype stability, only cells at passages 2–3 were used. Cells were divided into six groups: Blank group, OA group, MT group, MT + CsA group, MT + DMSO group, and MT + 740Y‐P group. OA modeling was established by stimulating chondrocytes with 10 ng/mL IL‐1β (HY‐P7097, MCE) for 24 h. Cells were subsequently treated with MT (10 ng/mL), CsA (2 μM), and equivalent DMSO or 740Y‐P (30 μM) for 6‐h co‐incubation [20].

### 2.8 Cell Counting Kit‐8 (CCK‐8) Assay

Cell viability was assessed using the CCK‐8 assay (C0039, Beyotime Shanghai, China). Briefly, cells from each treatment group were incubated with 10 μL/well CCK‐8 solution in the dark for 2 h. Absorbance was measured at 450 nm using a microplate reader (Varioskan LUX, Thermo Scientific).

### 2.9 Flow Cytometry

Cell apoptosis was evaluated using the Annexin V‐fluorescein isothiocyanate/propidium iodide (Annexin V‐FITC/PI) double‐staining cell assay kit (BB‐4101‐50 T, BestBio, Shanghai, China). Cells were washed twice with phosphate‐buffered saline (PBS) by centrifugation (500 g, 4°C, 5 min), and resuspended in 400 μL of 1× Annexin V binding solution. Annexin V‐FITC staining solution (5 μL) was added, and cells were incubated for 15 min at 2°C–8°C in the dark. PI staining solution (5 μL) was then added, and samples were immediately analyzed by flow cytometry (CytoFLEX, Beckman Coulter, Brea, CA, USA).

### 2.10 Transmission Electron Microscopy (TEM) Detection of Mitochondrial Structure

Mitochondrial ultrastructure was examined by TEM. Cells were collected, centrifuged, and fixed in electron microscope fixative solution (CR2206151, Servicebio, Wuhan, Hubei, China). Following fixation, cells were dehydrated in a graded ethanol series, embedded, sectioned, and imaged using a TEM (HT7800, Hitachi, Tokyo, Japan).

### 2.11 JC‐1 Detection of Mitochondrial Membrane Potential (MMP)

MMP was measured using the JC‐1 assay kit (C2006, Beyotime). Briefly, 1 mL of JC‐1 staining working solution and 1 mL of culture medium were added to each well, and cells were incubated at 37°C for 20 min. A 1× JC‐1 staining buffer was prepared by diluting 5× stock with distilled water (4:1) and kept on ice. After incubation, the supernatant was discarded, and cells were rinsed twice with JC‐1 staining buffer (1×), resuspended in 2 mL fresh medium, and analyzed with a fluorescence microplate reader (Varioskan LUX). JC‐1 monomers were detected at 490/530 nm (excitation/emission), and aggregates at 525/590 nm. The fluorescence ratio of aggregates to monomers was used as the index of MMP.

### 2.12 Autophagosome and Mitochondrial Immunofluorescence Co‐Localization

Co‐localization of mitochondria with microtubule‐associated protein 1 light chain‐3B (LC3B) is generally regarded as an indicator of mitophagy. Cells were cultured on confocal plates and processed according to the experimental design. After discarding the culture medium, cells were incubated with Mito‐Tracker Green (#9074, 100 nM, CST, Danvers, MA, USA) and LC3B Red (ab225383, 1:1000, Abcam, Cambridge, UK) at 37°C for 15 min. Nuclei were counterstained with 4′,6‐Diamidino‐2‐Phenylindole for 5–10 min, followed by three PBS washes. Images were acquired using a confocal microscope (LCS‐SP8‐STED, Leica, Wetzlar, Germany). Co‐localized particles (positive for both LC3B and Mito‐Tracker) were quantified using ImageJ software (National Institutes of Health, Bethesda, MD, USA). Six random visual fields were selected per group, and the mean number of co‐localized particles per cell was counted.

### 2.13 Western Blot

Samples were lysed in radio‐immunoprecipitation assay buffer (P0013B, Beyotime), and total proteins were extracted using a protein extraction kit (P0027, Beyotime). Protein concentrations were determined with the bicinchoninic acid assay (P0011, Beyotime). Equal amounts of protein (20 μg per lane) were separated on SDS–polyacrylamide gels and transferred to 0.45 μm polyvinylidene fluoride (PVDF) membranes. Membranes were blocked in 5% skim milk at room temperature for 1 h and incubated overnight at 4°C with primary antibodies against: rabbit anti‐PTEN‐induced putative kinase 1 (PINK1) (1:1000, ab216144, Abcam), Parkin (1:2000, PA5‐13399, Invitrogen, Carlsbad, CA, USA), LC3B (1:2000, ab192890, Abcam), p62 (1:10000, ab109012, Abcam), PI3K (1:1000, #4249, CST), phosphorylated (p)‐PI3K (1:1000, #4228, CST), p‐AKT (1:3000, 28731–1‐AP, Proteintech, Wuhan, Hubei, China), AKT (1:5000, 10176–2‐AP, Proteintech), FoxO3 (1:5000, 10849–1‐AP, Proteintech), COL2A1 (ab307674, 1:1000, abcam), MMP3 (ab52915, 1:5000, abcam), MMP13 (ab39012, 1:3000, abcam), and ADAMTS4 (ab314856, 1:1000, abcam). After washing, membranes were incubated with horseradish peroxidase‐labeled goat anti‐rabbit immunoglobulin G (1:5000, ab205714, Abcam) for 1 h at room temperature. Protein bands were visualized with chemiluminescence reagents (Thermo Scientific), and grayscale densitometry was performed. Glyceraldehyde‐3‐phosphate dehydrogenase (1:3000, ab8245, Abcam) served as the internal control.

### 2.14 Bioinformatics Analysis

Potential targets were identified by searching “Osteoarthritis, Melatonin, and Mitophagy” in the GeneCards database (https://www.genecards.org/). Intersecting targets were determined using the Draw Venn Diagram tool (http://bioinformatics.psb.ugent.be/webtools/Venn/). A protein–protein interaction (PPI) network of melatonin‐ and OA‐related mitophagy targets was generated using the STRING11.5 database (https://cn.string‐db.org/), and visualized in Cytoscape3.9.2 software (developed by the Institute for Systems Biology, University of California San Diego, University of California San Francisco, and Pasteur Institute). Gene Ontology (GO) enrichment analysis was performed using R software, focusing on cellular components, biological processes, and molecular functions. Kyoto Encyclopedia of Genes and Genomes (KEGG) pathway enrichment analysis was conducted using the DAVID Database (https://david.ncifcrf.gov), and the results were visualized with R. Pathway maps were compared with the KEGG database (https://www.kegg.jp/).

### 2.15 Statistical Analysis

All data analyses and plots were performed using GraphPad Prism 9.5 (GraphPad Software Inc., San Diego, CA, USA). All data analyses and plots were performed using the Kolmogorov–Smirnov test. Results were expressed as mean ± standard deviation (SD). Differences between two groups were analyzed using independent sample *t* tests. One‐way analysis of variance (ANOVA) followed by Tukey's multiple comparisons test was applied for multi‐group analyses. All *p*‐values were two‐tailed, and statistical significance was set as *p* < 0.05.

## 3 Results

### 3.1 MT Promoted Mitophagy in Chondrocytes of OA Rats, Thereby Reducing Their Apoptosis

**FIGURE 1:**
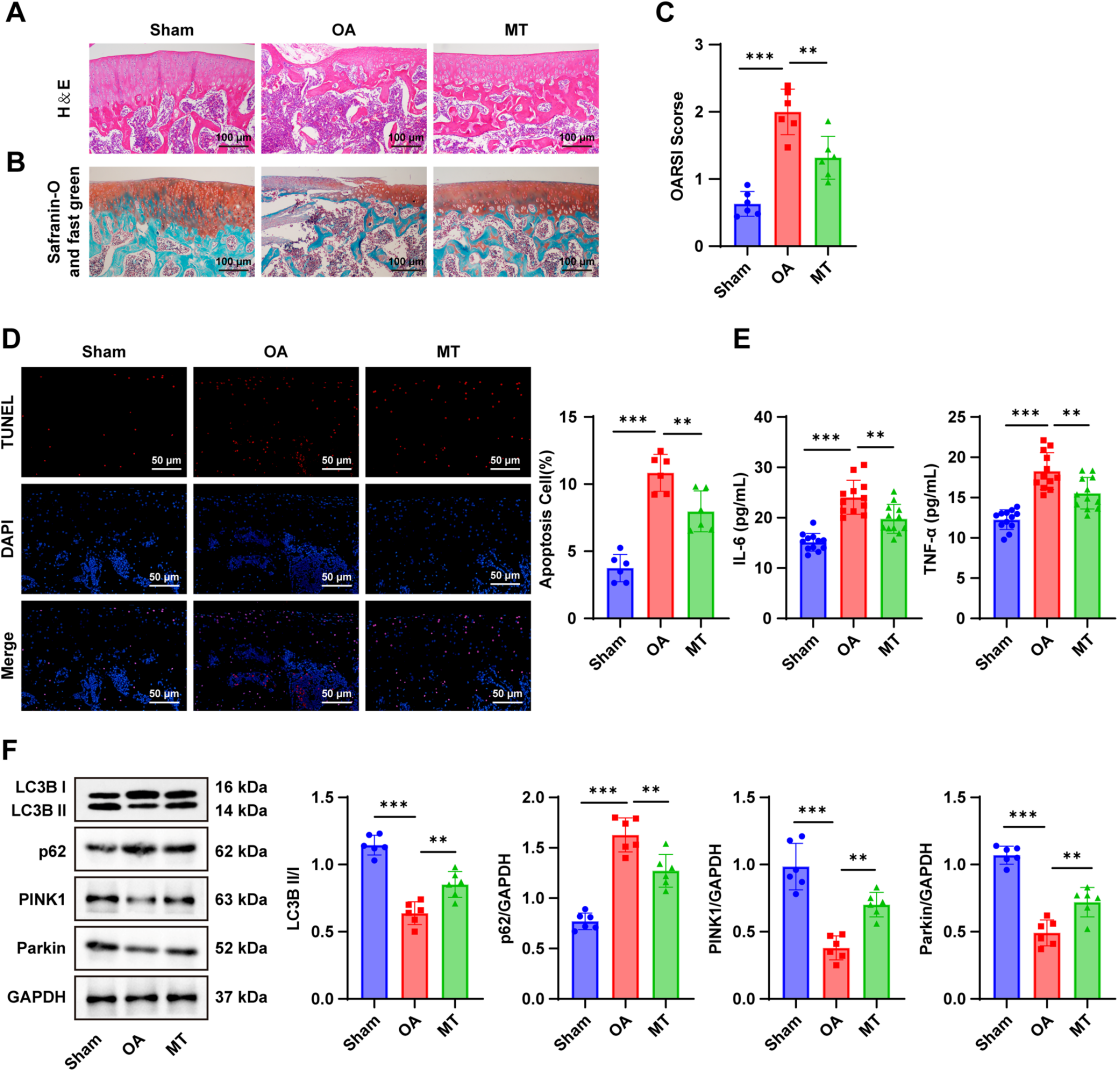
MT promoted mitophagy in chondrocytes of OA rats, thereby reducing apoptosis. (A) H&E staining to observe cartilage tissue pathology (*n* = 6); (B) Safranin O‐fast green staining to examine cartilage pathological changes (*n* = 6); (C) OARSI scoring to evaluate the progression of OA (*n* = 6); (D) TUNEL staining to evaluate chondrocyte apoptosis in cartilage tissues (*n* = 6); (E) ELISA to measure levels of inflammatory indicators IL‐6 and TNF‐α in synovial fluid (*n* = 12); (F) Western blot to determine mitophagy markers PINK1, Parkin, LC3‐II, LC3‐I, and p62 (*n* = 6). Data were presented as mean ± SD. Data comparisons among multiple groups were performed using one‐way ANOVA, followed by Tukey's multiple comparison test. The *p* value was obtained from a two‐sided test. **p < 0.01, ***p < 0.001.

To investigate the role of MT in OA, an OA rat model using ACLT was used, followed by MT treatment. H&E staining revealed that compared with the Sham group, rats in the OA group exhibited incomplete joint cartilage structure, a rough surface, a narrowed joint space, and disorganized trabecular bone (Figure 1A). Safranin O‐fast green staining showed partial ECM loss and uneven staining (Figure 1B) in OA cartilage, accompanied by significantly higher OARSI scores (*p* < 0.01, Figure 1C). TUNEL staining demonstrated increased chondrocyte apoptosis in the OA group compared with the Sham group (*p* < 0.01, Figure 1D). ELISA results showed elevated levels of IL‐6 and TNF‐α in synovial fluid from OA rats (all *p* < 0.01, Figure 1E). These findings confirmed the successful establishment of the OA model.

Abnormal mitophagy plays a crucial role in OA [6]. Western blot analysis demonstrated decreased protein levels of PINK1 and Parkin, and a reduced LC3B II/I ratio in OA cartilage, whereas p62 protein level was elevated (all *p* < 0.01, Figure 1F), indicating impaired mitophagy. MT treatment improved cartilage morphology, restored joint space, and preserved ECM integrity, with more uniform staining. MT also significantly reduced OARSI scores, decreased apoptosis, lowered IL‐6 and TNF‐α levels, increased PINK1 and Parkin expression, raised the LC3B II/I ratio, and reduced p62 protein levels (all *p* < 0.01, Figure 1A–F). Collectively, these results suggest that MT enhanced mitophagy in chondrocytes of OA rats, thereby mitigating apoptosis.

### 3.2 Inhibition of Mitophagy Partially Reversed the Improvement Mediated by MT in OA Rats

**FIGURE 2:**
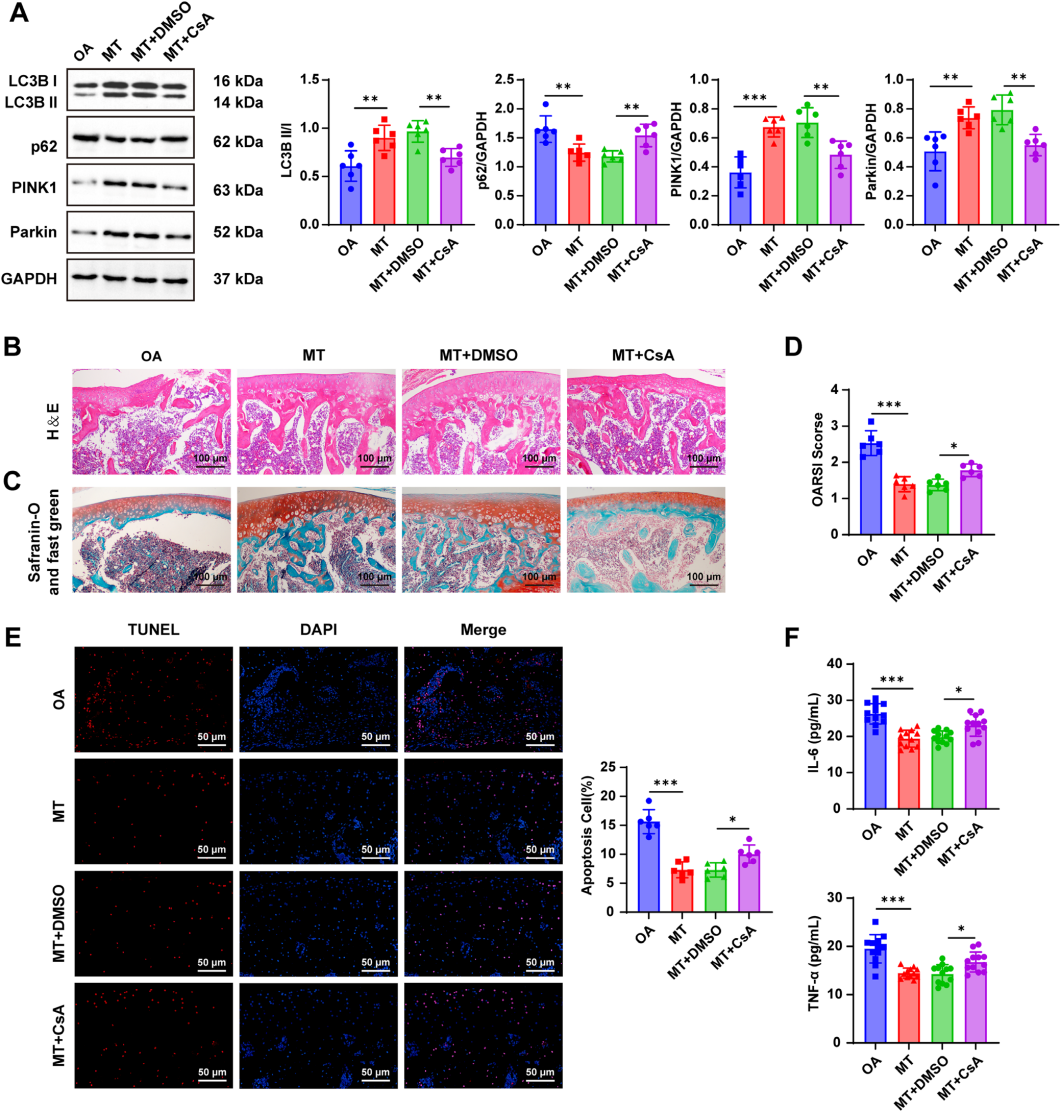
Mitophagy inhibition partially nullified the improvement effect of MT on OA rats. (A) Western blot detection of mitophagy markers PINK1, Parkin, LC3‐II, LC3‐I, and p62 (*n* = 6); (B) H&E staining to observe cartilage tissue pathology (*n* = 6); (C) Safranin O‐fast green staining to observe cartilage pathological changes (*n* = 6); (D) OARSI scoring to assess the progression of OA (*n* = 6); (E) TUNEL staining to evaluate cell apoptosis in cartilage tissues (*n* = 6); (F) ELISA to examine levels of inflammatory indicators IL‐6 and TNF‐α in synovial fluid (*n* = 12). Data were denoted as mean ± SD. Data comparisons among multiple groups were conducted using one‐way ANOVA, followed by Tukey's multiple comparison test. The *p* value was obtained from a two‐sided test. *p < 0.05, **p < 0.01, ***p < 0.001.

To confirm the involvement of mitophagy, OA rats were treated with CsA (a mitophagy inhibitor) and MT. Co‐treatment reduced PINK1 and Parkin expression and the LC3B II/I ratio, while elevating p62 protein levels (all *p* < 0.01, Figure 2A). Histological analyses showed incomplete cartilage, rough joint surfaces, narrowed cavities, disorganized trabeculae, and heterogeneous ECM staining in the MT + CsA group (Figure 2B,C). These rats also exhibited higher OARSI scores (*p* < 0.05, Figure 2D), increased apoptosis (*p* < 0.05, Figure 2E), and elevated IL‐6 and TNF‐α in synovial fluid (all *p* < 0.05, Figure 2F). These findings indicate that inhibition of mitophagy attenuated the beneficial effects of MT, underscoring mitophagy as a key mechanism of MT‐mediated cartilage protection.

### 3.3 MT Regulated IL‐1β‐Induced Chondrocyte Apoptosis and ECM Degradation by Promoting Mitophagy

**FIGURE 3:**
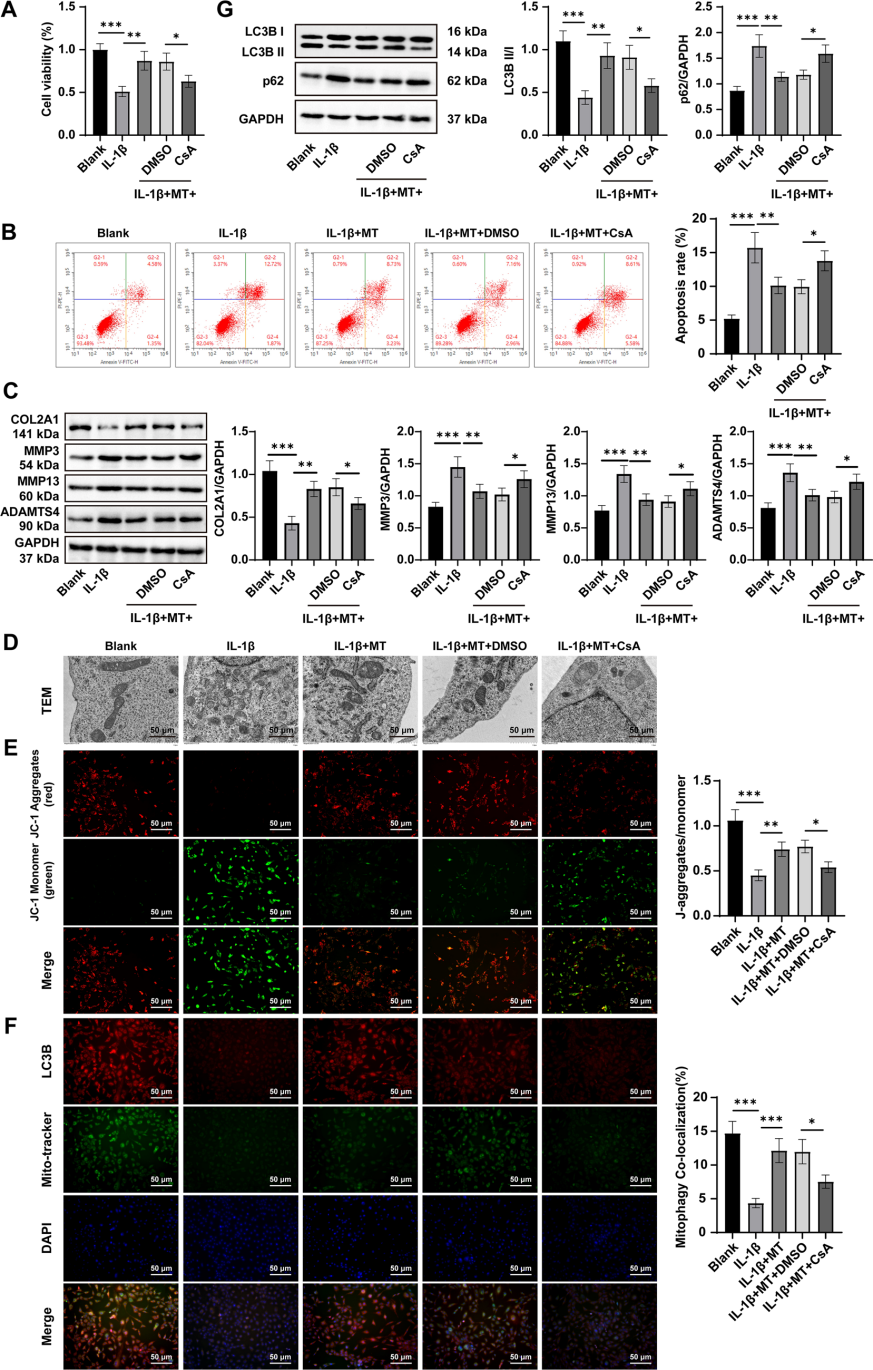
MT regulated IL‐1β‐induced chondrocyte apoptosis and ECM degradation by promoting mitophagy. (A) CCK‐8 assay to assess cell viability; (B) Flow cytometry to evaluate cell apoptosis; (C) Western blot analysis was conducted to assess the expression of extracellular matrix–related proteins, including COL2A1, MMP3, MMP13, and ADAMTS4; (D) Mitochondrial morphological changes observed under TEM; (E) The JC‐1 assay to measure MMP; (F) Immunofluorescence to detect the mitophagy level (Mito‐Tracker+LC3B+); (G) Western blot to measure autophagy markers LC3‐II, LC3‐I, and p62. Cell experiments were independently repeated three times. Data were presented as mean ± SD. One‐way ANOVA was utilized for multi‐group comparisons, and the post hoc test was performed using Tukey's multiple comparison test. The *p* value was obtained from a two‐sided test. *p < 0.05, **p < 0.01, ***p < 0.001.

To further validate these findings in vitro, primary chondrocytes were stimulated with IL‐1β for 24 h. CCK‐8 results showed significantly reduced viability (*p* < 0.05, Figure 3A), while flow cytometry demonstrated increased apoptosis (*p* < 0.05, Figure 3B). Western blot analysis revealed decreased COL2A1 expression and increased MMP3, MMP13, and ADAMTS4 expression after IL‐1β stimulation (*p* < 0.05, Figure 3C). These findings indicate that the in vitro cell model was successfully established. TEM analysis showed mitochondrial swelling, vacuolization, and structural disruption (Figure 3D). JC‐1 staining indicated a marked loss of MMP (*p* < 0.05, Figure 3E). Immunofluorescence analysis showed reduced mitophagy (Mito‐Tracker+LC3B+) in chondrocytes exposed to IL‐1β induction (*p* < 0.01, Figure 3F). Consistently, western blot demonstrated a decreased LC3B II/I ratio and elevated p62 protein levels after IL‐1β induction (all *p* < 0.05, Figure 3G). When chondrocytes were co‐treated with MT during IL‐1β stimulation, mitochondrial density and structural integrity were restored, MMP was preserved, mitophagy was enhanced, the LC3B II/I ratio increased, and p62 expression decreased. These changes were accompanied by improved cell viability, maintenance of ECM, and reduced apoptosis (all *p* < 0.05, Figure 3A–G). However, the addition of the mitophagy inhibitor CsA reversed these protective effects: mitophagy, cell viability, and ECM integrity declined, while apoptosis increased (all *p* < 0.05, Figure 3A–G). Together, these findings indicate that MT mitigates IL‐1β‐induced chondrocyte apoptosis and ECM degradation by enhancing mitophagy.

### 3.4 Bioinformatics Analysis of the Molecular Mechanism of MT Regulating Mitophagy in OA

**FIGURE 4:**
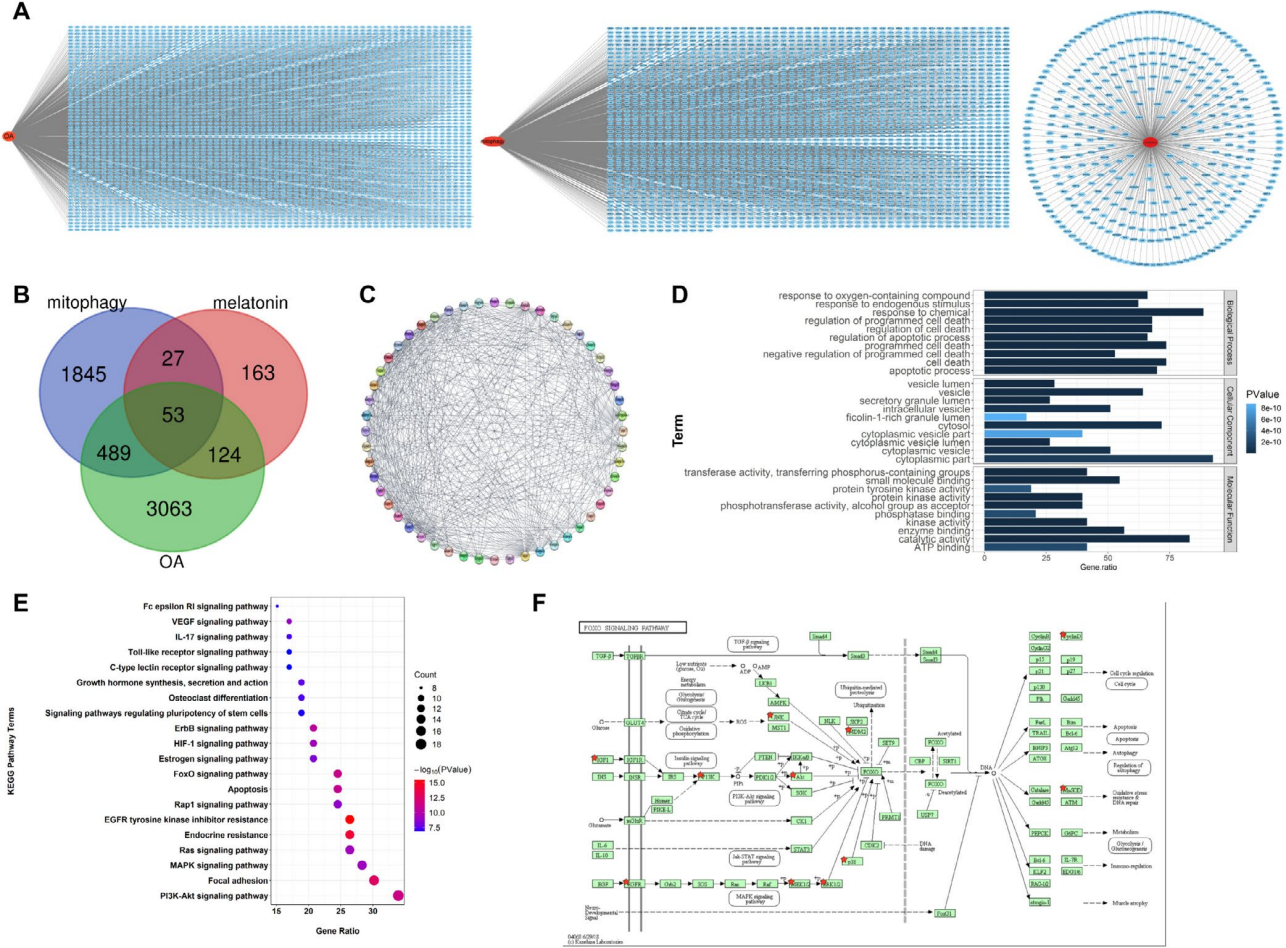
The connection between MT and mitophagy‐related functions in OA based on data from the gene database. (A) Display diagram of targeted genes for OA, mitophagy, and MT in GeneCards database; (B) Venn diagram showing the intersection of the three target gene results predicted by the GeneCards database; (C) PPI network of MT and mitophagy‐related proteins in OA; (D) The GO analysis on MT and mitophagy‐related proteins in OA; (E) The KEGG analysis on MT and mitophagy‐related proteins in OA; (F) The FOXO pathway diagram.

To further explore the molecular mechanism by which MT promotes mitophagy in OA, we used the GeneCards database to identify target genes of OA, mitophagy, and MT. A total of 3729 OA‐related genes, 2414 mitophagy‐related genes, and 367 MT‐related genes were retrieved (Figure 4A) (Tables [Link], [Link], [Link]). Intersection analysis with the Draw Venn Diagram identified 53 overlapping genes (Figure 4B,C). The GO enrichment analysis revealed that these 53 common overlapping genes were predominantly associated with biological processes such as programmed cell death, response to environmental stimuli, and oxidative stress (Figure 4D). KEGG pathway indicated that 20 pathways were potentially involved in MT‐mediated promotion of mitophagy in OA (Figure 4E). Among these, the FOXO pathway showed the highest overlap with the common genes, alongside pathways related to apoptosis, autophagy, and oxidative stress (Figure 4F). Previous studies have reported that MT regulates mitochondrial redox homeostasis and autophagy via the FoxO3 pathway to protect chondrocytes [21] and that MT can modulate the PI3K‐AKT‐FoxO3 pathway [22]. Furthermore, coenzyme Q10 has been shown to enhance FoxO3‐mediated autophagy by inhibiting PI3K/AKT signaling, thereby suppressing RANKL‐induced osteoclastogenesis [23]. Taken together, these findings suggest that the PI3K/AKT/FoxO3 pathway may represent a key mechanism through which MT promotes mitophagy and alleviates OA.

### 3.5 MT Promoted Mitophagy by Increasing FoxO3 Expression Through the PI3K/AKT Pathway, Thereby Inhibiting Chondrocyte Apoptosis

**FIGURE 5:**
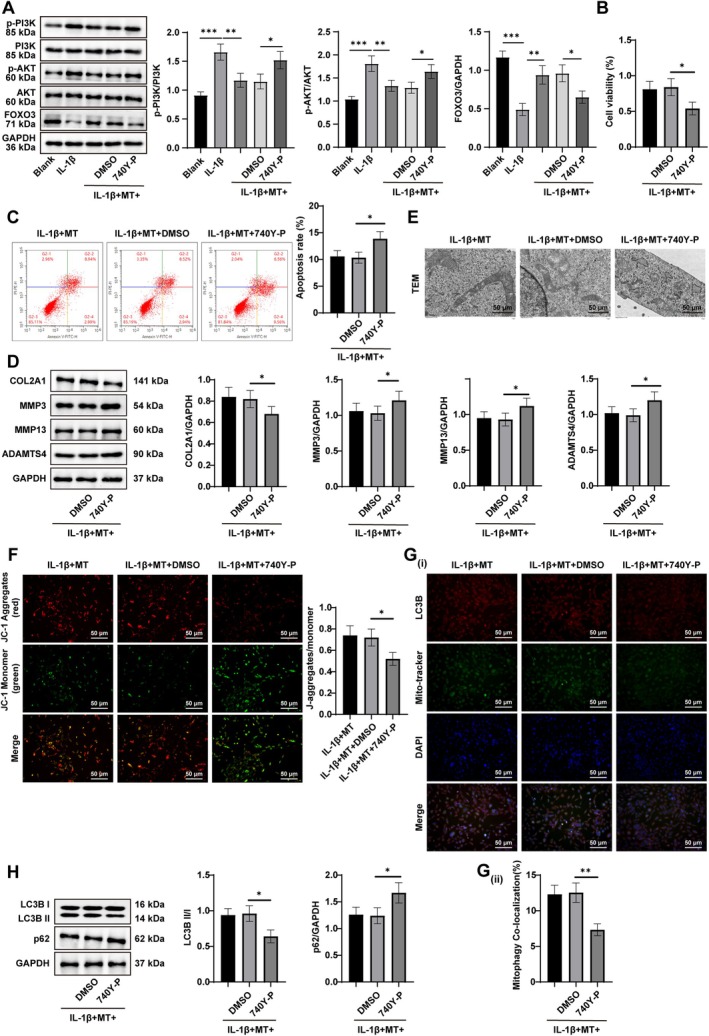
MT increased FoxO3 expression to boost mitophagy via the PI3K/AKT pathway, thus curbing chondrocyte apoptosis. (A) Assessment of PI3K, p‐PI3K, AKT, p‐AKT, and FoxO3 expression by western blot; (B) Evaluation of cell viability by CCK‐8 assay; (C) Evaluation of cell apoptosis by flow cytometry; (D) Western blot analysis was conducted to assess the expression of extracellular matrix–related proteins, including COL2A1, MMP3, MMP13, and ADAMTS4; (E) Observation of mitochondrial morphological changes using TEM; (F) Examination of MMP by the JC‐1 assay; (G) Assessment of mitophagy (Mito Tracker+LC3B+) using immunofluorescence; (H) Determination of autophagy markers LC3‐II, LC3‐I, and p62 by western blot. Cell experiments were repeated thrice independently. Data were expressed as mean ± SD. One‐way ANOVA was used to compare the data among multiple groups. Tukey's multiple comparison test was used for the post hoc test. The *p* value was obtained from a two‐sided test. *p < 0.05, **p < 0.01, ***p < 0.001.

To validate this hypothesis, western blot detection was performed. Following IL‐1β stimulation, chondrocytes exhibited increased p‐PI3K/PI3K and p‐AKT/AKT ratios, along with reduced FoxO3 expression. Co‐treatment with MT reversed changes, significantly decreasing PI3K/AKT phosphorylation and restoring FoxO3 expression (all *p* < 0.05, Figure 5A). When the PI3K activator 740Y‐P was added together with MT and IL‐1β, PI3K/AKT signaling was re‐activated, resulting in decreased FoxO3 expression, impaired mitochondrial density, reduced MMP, diminished mitophagy (Mito‐Tracker+LC3B+), and a lower LC3B II/I ratio. Additionally, chondrocytes exhibited increased p62 expression, reduced viability, impaired ECM integrity, and increased apoptosis (all *p* < 0.05, Figure 5A–H). These findings indicate that MT promotes mitophagy by inhibiting PI3K/AKT signaling, thereby upregulating FoxO3 expression. This enhanced mitophagy ultimately protects chondrocytes from apoptosis and preserves cartilage integrity.

### 3.6 MT Enhanced Mitophagy to Reduce Apoptosis of Cartilage Tissue Cells Through the PI3K/AKT/FoxO3 Pathway, Thereby Improving OA

**FIGURE 6:**
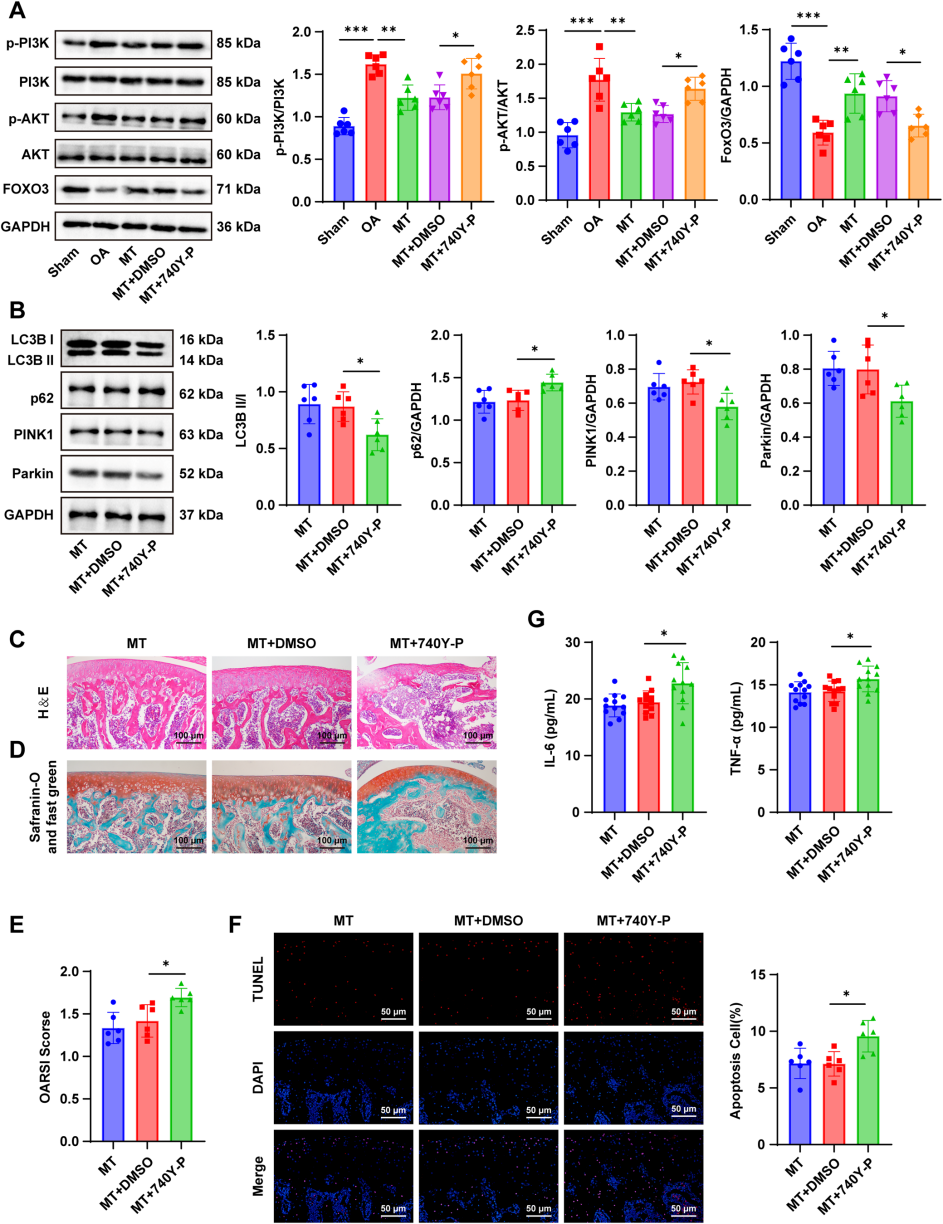
MT enhanced mitophagy to inhibit chondrocyte apoptosis in OA rats via the PI3K/AKT/FoxO3 pathway. (A) The western blot analysis on PI3K, p‐PI3K, AKT, p‐AKT, and FoxO3 expression; (B) Mitophagy markers PINK1, Parkin, LC3‐II, LC3‐I, and p62 assessed by western blot (*n* = 6); (C) Observation of cartilage tissue pathology using H&E staining; (D) Observation of cartilage pathology changes using safranin O‐fast green staining; (E) Evaluation of OA progression using OARSI scores (*n* = 6); (F) Assessment of chondrocyte apoptosis by TUNEL staining (*n* = 6); (G) Examination of inflammatory indicators IL‐6 and TNF‐α in synovial fluid by ELISA (*n* = 12). Data were expressed as mean ± SD. One‐way ANOVA was utilized for multi‐group comparisons, followed by Tukey's multiple comparison test. The *p* value was obtained from a two‐sided test. *p < 0.05, **p < 0.01, ***p < 0.001.

To validate the role of the PI3K/AKT/FoxO3 pathway in vivo, OA rats were treated with the PI3K activator 740Y‐P in combination with MT. Western blot analysis showed that compared with the Sham group, OA rats exhibited elevated p‐PI3K/PI3K and p‐AKT/AKT ratios and reduced FoxO3 expression in cartilage tissue. MT treatment reversed these effects, decreasing PI3K and AKT phosphorylation while increasing FoxO3 expression (all *p* < 0.05, Figure 6A). However, co‐administration of MT and 740Y‐P re‐activated PI3K/AKT signaling, which was accompanied by reduced FoxO3 expression, decreased PINK1 and Parkin protein levels, and a lower LC3B II/I ratio, and increased p62 expression (all *p* < 0.05; Figure 6A). Histological evaluation further revealed that MT + 740Y‐P treatment led to disrupted cartilage architecture, including rough articular surfaces, narrowed joint space, disorganized trabeculae, partial ECM loss, and uneven staining (all *p* < 0.05, Figure 6B–G). Taken together, these findings demonstrate that MT mitophagy inhibits PI3K/AKT signaling and upregulates FoxO3, thereby reducing apoptosis of cartilage cells and ameliorating OA pathology in vivo.

## 4 Discussion

OA is a chronic degenerative joint disease characterized by progressive cartilage breakdown, joint pain, and functional impairment [24]. Current treatment strategies primarily aim to relieve pain and manage symptoms, with minimal influence on disease progression [25]. Given the multifaceted biological properties of melatonin (MT), including antioxidative, analgesic, anti‐apoptosis, and anti‐inflammatory effects, this study employed bioinformatics, in vivo, and in vitro experiments to investigate its protective role in OA. Our findings demonstrate that MT alleviates OA by reducing chondrocyte apoptosis through activation of mitophagy via the PI3K/AKT/FoxO3 pathway (see Figure 6).

Mitophagy serves as an essential protective mechanism by removing damaged mitochondria, thereby reducing oxidative stress, limiting chondrocyte apoptosis, and slowing OA progression [8]. OA‐associated inflammation triggers mitochondrial injury and activates the PINK1/Parkin‐mediated pathway. PINK1 accumulates on impaired mitochondria, recruits Parkin, and promotes the formation of the Parkin–Ub complex. Through the adaptor protein P62, this complex links with LC3B and targets mitochondria to autophagosomes [26]. Impaired Parkin expression, as observed in IL‐1β‐stimulated OA chondrocytes, compromises its recruitment to P62 and aggravates apoptosis [8]. LC3B, which exists as cytoplasmic LC3B‐I and membrane‐bound LC3B‐II, is a key autophagy marker. LC3B‐I, a soluble cytoplasmic form, conjugates with phosphatidylethanolamine to form LC3B‐II, which assists in autophagosome membrane binding during autophagy [27]. Reduced PINK1 and Parkin expression in OA chondrocytes, together with elevated P62 and a decreased LC3B‐II/LC3B‐I ratio, indicate impaired autophagosome formation [28]. In line with these reports, our ACLT‐induced OA rats exhibited ECM loss, increased OARSI scores, elevated apoptosis, higher IL‐6 and TNF‐α levels, and abnormal mitophagy characterized by decreased PINK1, Parkin, and LC3B II/I ratio, and increased p62 expression.

Previous studies have reported analgesic and cytoprotective effects of MT in various conditions such as fibromyalgia, neuropathic pain, chronic headache, and burn‐related pain, with good safety and tolerability [29]. Clinical evidence further supports its therapeutic potential in OA‐associated pain [30]. Mechanistically, MT has been shown to upregulate PINK1, Parkin, and LC3 II proteins while reducing p62 in cardiomyocytes [31], consistent with its mitophagy‐inducing properties. By removing dysfunctional mitochondria, mitophagy reduces ROS production and inhibits apoptosis, thereby slowing OA progression [32]. Our data corroborate these findings: MT promoted mitophagy in OA rats and IL‐1β‐stimulated chondrocytes, reducing apoptosis and preserving ECM integrity. Importantly, inhibition of mitophagy with CsA attenuated these protective effects, underscoring the essential role of mitophagy. IL‐1β, a key pro‐inflammatory cytokine, promotes ECM degradation and joint inflammation, accelerating OA progression [33]. In in vitro tests, MT enhanced mitophagy, boosted chondrocyte viability, and inhibited apoptosis in IL‐1β‐treated cells, while CsA reversed these effects. These findings confirm that MT protects chondrocytes from IL‐1β‐induced apoptosis and ECM degradation by inducing mitophagy.

Bioinformatics analysis further suggested that the PI3K/AKT/FoxO3 pathway is a critical regulator of MT‐mediated mitophagy. Suppression of the PI3K/AKT pathway enhances autophagy in chondrocytes by blocking mTOR activation, thereby mitigating OA [34]. FoxO3, a master regulator of cellular homeostasis, promotes both autophagy and mitophagy [35]. PI3K/AKT signaling negatively regulates FoxO3 activity, inhibiting its transcriptional function [36]. Consistent with these mechanisms, IL‐1β stimulation increased PI3K/AKT phosphorylation and decreased FoxO3 expression in chondrocytes, while MT reversed these changes, thereby restoring mitophagy and reducing apoptosis [21]. In vivo, MT also reduced apoptosis in cartilage tissue through the same pathway, confirming the central role of PI3K/AKT/FoxO3 signaling.

In conclusion, our study demonstrated that MT promotes mitophagy through inhibition of the PI3K/AKT/FoxO3 pathway and upregulation of FoxO3, thereby reducing apoptosis in cartilage tissues and ameliorating OA. These findings highlight the therapeutic potential of MT in OA and provide a mechanistic basis for its application. Nevertheless, several limitations remain. This study did not include clinical samples for validation. Moreover, MT's effects are mediated via the MT1 or MT2 receptor, which may exert distinct influences. MT attenuates AKT/mTOR activation through MT2 receptor‐mediated modulation of PI3K, thereby enhancing autophagy [37]. Notably, MT1 expression is reduced in OA tissues compared with normal tissues, suggesting that MT2 may play a predominant role in MT‐mediated regulation of the PI3K/AKT/FoxO3 pathway [38]. However, the precise receptor‐specific mechanisms were not elucidated here. Future studies aim to confirm these findings in clinical samples, including small‐scale trials assessing pain, function, and radiographic progression, and further delineate receptor‐specific signaling in MT‐mediated mitophagy.

## Ethics Statement

All animal experiments in this study were reviewed and approved by the Medical Ethics Committee of The Second Affiliated Hospital of Harbin Medical University, approval number: YJSKY2022‐080. We rigorously followed the authorized protocol and conducted the animal experiments based on the minimized number of animals and their least pains.

## Conflicts of Interest

The authors declare no conflicts of interest.

## Supporting information


**Table S1:** kjm270131‐sup‐0001‐TableS1.xlsx.

**Table S2:** kjm270131‐sup‐0002‐TableS2.csv.

**Table S3:** kjm270131‐sup‐0003‐TableS3.xlsx.

## Data Availability

The data that support the findings of this study are available from the corresponding author upon reasonable request.
